# Income Inequality by Gini-Coefficient on Suicide Death in Iran: A Review of National Data

**Published:** 2019-08

**Authors:** Yousef VEISANI, Ali DELPISHEH, Reza VALIZADEH, Sattar KIKHAVANI

**Affiliations:** 1. Psychosocial Injuries Research Center, Ilam University of Medical Sciences, Ilam, Iran; 2. Department of Clinical Epidemiology, School of Public Health, Ilam University of Medical Sciences, Ilam, Iran; 3. Department of Psychology, Psychosocial Injuries Research Center, Ilam University of Medical Sciences, Ilam, Iran

**Keywords:** Gini-coefficients, Inequality, Concentration index, Suicide

## Abstract

**Background:**

Research in source of inequality and enhance of knowledge can be reducing the inequalities in the coming decades. Therefore, we aimed to ascertain effects of income inequality measured by Gini-coefficient to death from suicide in Iran.

**Methods:**

This is an ecological study on the relation of Gini-coefficient and suicide death in Iran. Data were obtained from Iranian Urban and Rural Household Income for Gini-coefficient and Expenditure Survey and Iranian Forensic Medicine Organization for suicide. Concentration Index was used to determine of inequality by Gini-coefficient in suicide death and prediction model was applied by Stata software. Significant level considered less than 5%.

**Results:**

A Gini-coefficient between 0.2523 and 0.3755 (mean, 0.3092) was observed. The overall concentration index CI was −0.10 (95% CI= −0.19 to −0.01), therefore our results confirmed a positive inequality in incidence suicide rate result from income inequality in Iran.

**Conclusion:**

Our results showed a positive inequality due to Gini-coefficients in suicide death. This study could be a start for investigation of inequality source in geographical units and at the individual level in all provinces

## Introduction

Suicide is a serious concern globally. About one million people die from suicide annually (1). Over the last half-century, a suicide rate has not declined and has increased by 60% of countries in the world. The incidence rate of suicide now 11.5 per 100,000 people, annually (2). The rate of suicide fluctuates in world 5 to 20 per 100,000. In Brazil were reported 5.8 per 100,000 people, meanwhile in Korea reaches to 21 per 100,000 people (3).

In Iran, the suicide incidence rate in 2015 was reported at 3.7 per 100,000 people (4). There are different risk factors that have been associated to suicide in Iran such as marital status and illiteracy (5) social inequalities (6), inequality in addiction and mental health (7) and male and lower educational level (8). The disparities in incidence rate of suicide have been shown in Iran in previous, which indicated that different factors should be affected by suicide in different reign (6, 9).

Research in source of inequality and enhance of knowledge can be reducing the inequalities in the coming decades. Effects of economic indexes such as Gini-coefficient on suicidal behavior have been acknowledged, but remain so as controversial subject. Some have found a deprived situation increasing the risk of suicide (10, 11), while others oppose it showed that converging of Gini-coefficient over time, not couple with reduce in suicide rates (12).

Therefore, we aimed to assess the inequality in incidence rate of suicide according to income inequality measured by Gini-coefficient in Iran.

## Materials and Methods

This is an ecological study on the relation of Gini-coefficient and suicide in Iran. Data obtained from results of the Iranian Urban and Rural Household Income and Expenditure Survey for Gini-coefficient (13) and the annual reports of Iranian Forensic Medicine Organization for suicide rate (14). We defined inequality in the suicide death according to the Gini-coefficient by using concentration index (CI) among provinces. CI is the cumulative percentage of suicide against the cumulative percentage of population, ranked by Gini-coefficient from highest (0.3755) to lowest (0.2523). By this ranking process, the highest Gini-coefficient score has had first order of among provinces. If there is no inequality in the distribution, CI is zero. The value of CI is between −1 to +1. The negative value is indicating that suicide is more concentrated in disadvantaged provinces and the positive value indicates that the suicide is concentrated among the rich provinces (according to highest to lowest of Gini-coefficient).

### Statistical analysis

We were used from linear regression model for predictions of suicide rate by Gini-coefficient. Concentration index was used to determine of inequality by Gini-coefficient in suicide death. We used the Stata software version 11.2 (Stata Corp, College Station, TX, USA) to perform all the analytical operations as well as Epi Info™ software for drawing maps. Significant level considered less than 5%.

## Results

**Fig. 1:**
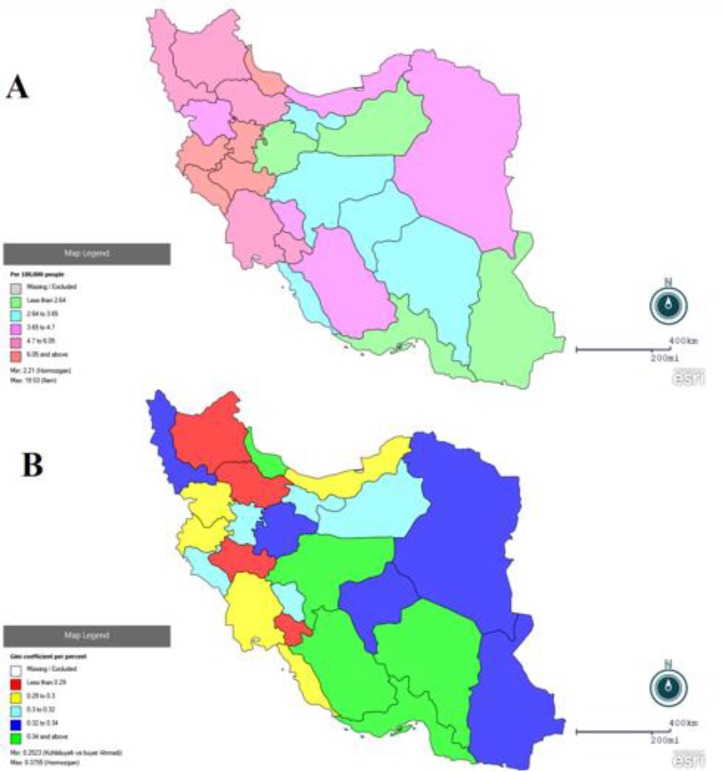
Distribution of Gini-coefficient (A) incidence suicide death rate (B) across the provinces in Iran

Figure 1 shows estimation of suicide rate per 100,000 and Gini-coefficient based on national data in Iran. A Gini-coefficient between 0.2523 and 0.3755 (mean, 0.3092) was observed, Kohgiluyeh and Boyer-Ahmad and Hormozgan had a lowest and highest rank (.2523 and .3755), respectively, and suicide rates were between 2.21 and 19.53 (mean, 5.13), the highest rate was observed in Ilam Province (19.53), and lowest rate in Hormozgan Province (2.21).

**Fig. 2:**
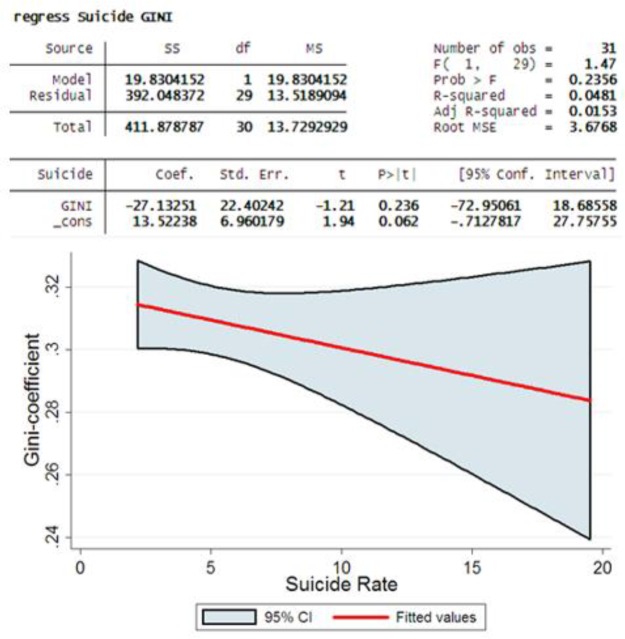
The relationship between incidence suicide death rate and Gini-coefficient in Iran

The results of prediction models by Gini-coefficient was showed that decline in Gini-coefficient was linked to decreasing trend in suicide death rate, but this association not supported by statically significant. (Coef=−27.13, *P*< 0.23) (Fig. 2).

**Fig. 3:**
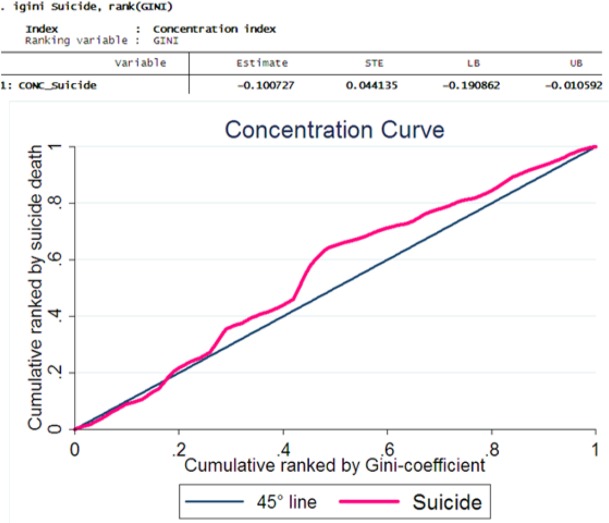
Concentration curves of incidence suicide death rate in Iran according to population ranked by Gini-coefficient

The curve of inequality was showed a positive inequality in suicide death rate by Gini-coefficient. The overall CI was −0.10 (95% CI = −0.19 to −0.01), therefore our results confirmed a positive inequality in incidence suicide rate result from income inequality in Iran (Fig. 3).

## Discussion

In this ecological method study, we have tried to ascertain income inequality effects, which measured by Gini-coefficient, in death from suicide in Iran. Our finding showed that death from suicide not equal in all Gini-coefficients and income inequality were having determinate in suicide death. Accordingly, in areas with lower incidence suicide death rate Gini-coefficients has the desire to diminish.

A result of current study is consistent with other studies that found significant relationship between having deprived economic status and suicide rates (15, 16). A significant relationship was reported between demographic factors such as educational level and suicide rate (18). Eastern Mediterranean region countries with low/ middle-income counterparts had higher suicide mortality rates compared to high-income countries (16).

Provinces with higher Gini-coefficients health disasters such as poorer mental health and limited access to health facilities. Therefore our results are line to studies that showed suicide rate is more common in disadvantaged population. Our finding suggested that reducing inequality can be one prevention program conducted in our country to handle of suicide.

In result by prediction model, decline in Gini-coefficient was related to decreasing trend in suicide death rate that is in line with Durkheim theories that proposed industrialization, population growth and urbanization has a socioeconomic change in suicide theories (17). A systematic review that conducted to relationship between socioeconomic factors and suicide showed that 27% of previous studies reported a direct association between education level and suicide and income and suicide 50% as well (18).

## Conclusion

We conducted ecological study and hypothesized that lower income by Gini-coefficient accompanied by higher incidence suicide death rate. There is a positive inequality due to Gini-coefficients in suicide death. Therefore, suicide rate more occurred in provinces that higher Gini-coefficients. This study could be a start for investigation of inequality source in geographical units and at the individual level in all provinces and whether is Gini-coefficient good predictor for suicidal behaviors?

## Ethical considerations

Ethical issues (Including plagiarism, informed consent, misconduct, data fabrication and/or falsification, double publication and/or submission, redundancy, etc.) have been completely observed by the authors.
